# Malaria control in Nepal 1963–2012: challenges on the path towards elimination

**DOI:** 10.1186/1475-2875-13-241

**Published:** 2014-06-23

**Authors:** Meghnath Dhimal, Bodo Ahrens, Ulrich Kuch

**Affiliations:** 1 Nepal Health Research Council (NHRC), Ministry of Health and Population Complex, Ramshah Path, Kathmandu, Nepal; 2 Biodiversity and Climate Research Centre (BiK-F), Senckenberg Gesellschaft für Naturforschung, Frankfurt am Main, Germany; 3 Institute for Atmospheric and Environmental Sciences, Goethe University, Frankfurt am Main, Germany; 4 Institute of Occupational Medicine, Social Medicine and Environmental Medicine, Goethe University, Frankfurt am Main, Germany

**Keywords:** *Anopheles*, Climate change, Cross-border, Environment, Insecticide, Imported malaria, Malaria elimination, *Plasmodium*, Resistance, Vector

## Abstract

**Background:**

Malaria is still a priority public health problem of Nepal where about 84% of the population are at risk. The aim of this paper is to highlight the past and present malaria situation in this country and its challenges for long-term malaria elimination strategies.

**Methods:**

Malariometric indicator data of Nepal recorded through routine surveillance of health facilities for the years between 1963 and 2012 were compiled. Trends and differences in malaria indicator data were analysed.

**Results:**

The trend of confirmed malaria cases in Nepal between 1963 and 2012 shows fluctuation, with a peak in 1985 when the number exceeded 42,321, representing the highest malaria case-load ever recorded in Nepal. This was followed by a steep declining trend of malaria with some major outbreaks. Nepal has made significant progress in controlling malaria transmission over the past decade: total confirmed malaria cases declined by 84% (12,750 in 2002 *vs* 2,092 in 2012), and there was only one reported death in 2012. Based on the evaluation of the National Malaria Control Programme in 2010, Nepal recently adopted a long-term malaria elimination strategy for the years 2011–2026 with the ambitious vision of a malaria-free Nepal by 2026. However, there has been an increasing trend of *Plasmodium falciparum* and imported malaria proportions in the last decade. Furthermore, the analysis of malariometric indicators of 31 malaria-risk districts between 2004 and 2012 shows a statistically significant reduction in the incidence of confirmed malaria and of *Plasmodium vivax,* but not in the incidence of *P. falciparum* and clinically suspected malaria.

**Conclusions:**

Based on the achievements the country has made over the last decade, Nepal is preparing to move towards malaria elimination by 2026. However, considerable challenges lie ahead. These include especially, the need to improve access to diagnostic facilities to confirm clinically suspected cases and their treatment, the development of resistance in parasites and vectors, climate change, and increasing numbers of imported cases from a porous border with India. Therefore, caution is needed before the country embarks towards malaria elimination.

## Background

**Figure 1:**
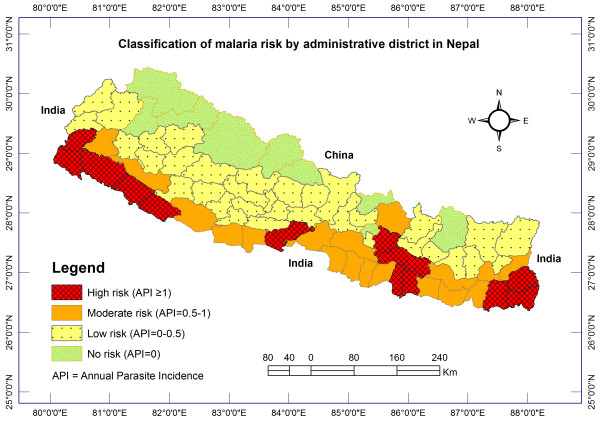
Classification of malaria risk districts in Nepal.

In Nepal, about 84% (23 million) of the people were at risk of malaria in 2012 with 4% at high risk. One million people live in areas with a reported incidence of more than one case per 1,000 population per year [1]. However, the scale of preventive interventions appears to have been limited in Nepal [2]. In recent years, malaria control activities have been carried out in 65 districts at risk out of 75 administrative districts [3]. In 2010, these 65 districts were further categorized for malaria control programme interventions. Based on the annual parasite incidence (API), there were 13 high-risk districts (API ≥1), 18 moderate-risk districts (API = 0.5-1), 34 low-risk districts (API = 0-0.5) and ten no-risk districts (API = 0) [3,4], as shown in Figure 1. The Global Fund to fight AIDS, tuberculosis and malaria (GFATM) started supporting a malaria control programme in high-priority, malaria-risk districts in Nepal in April 2004 [5]. Since 2011, the GFATM has scaled up its support for the malaria control programme in 18 additional moderate-risk districts [3,4]. The GFATM support is utilized for rapid diagnostic test (RDT) kits, artemisinin combination therapy (ACT), long-lasting insecticidal nets (LLINs), and information, education and communication/behaviour change communication (IEC/BCC) for LLIN use [4]. After the introduction of these interventions, the number of confirmed malaria cases in Nepal declined substantially.

Based on recommendations from the internal and external evaluation of Nepal’s malaria control programme in 2010, the country has been preparing for a pre-elimination phase since 2011. It has recently adopted a long-term malaria elimination strategy with the ambitious vision of a malaria-free Nepal by the year 2026 [3,4,6]. However, relatively little attention has been given to systematic analyses of the existing malaria data that are collected by the routine health surveillance system of the country. Moreover, there has not been any review of malaria in Nepal that addresses the epidemiological and entomological aspects of malaria transmission. Reviews of lessons from a country’s malaria control history help to develop flexible strategies for achieving malaria elimination goals [7]. Hence, this paper aims to highlight the past milestones, the present malaria situation in Nepal, and the challenges for future prevention and control strategies that need to be mastered to pave the way towards malaria elimination in this country.

## Methods

### Study area

Nepal is a mountainous country with highly diverse topography ranging from the lowland *terai* region (about 60 m above sea level) to the highest peak of the planet, Mount Everest (8,850 m). As a consequence, almost all types of climate from subtropical to alpine exist in the country [8]. Its 147,181 sq km of territory are situated on the southern slope of the central Himalayas, landlocked between China in the north and India in the east, south and west. Geographically, Nepal is broadly divided into three ecological regions: terai, hills and mountains, and administratively in five development regions and 75 districts. According to the latest census (2011), the total population of the country is 26,494,504 and has a growth rate of 1.35% per year. About half of the population (50.3%) live in the terai and 43 and 6.7% in the hill and mountain regions, respectively [9]. However, in terms of area, the mountains cover 35%, the hills 42% and the terai only 23% of the country. Thus, the terai has the highest population density (392 per sq km) and the mountains the lowest (34 per sq km) [9]. The majority of people (83%) in Nepal live in rural areas and only 17% in urban areas [9], and the number of reported malaria cases is higher in rural areas.

### Research design

A retrospective study was conducted to describe the trend of malaria of the last 50 years (1963–2012) and assess changes in malariometric indicators of the high- and moderate-risk districts after the introduction of the GFAMT programme (2004–2012) in Nepal.

### Data collection

Routine national health facility surveillance data of malariometric indicators were obtained from the Epidemiology and Disease Control Division (EDCD), Department of Health Services (DoHS), Government of Nepal. In Nepal, the national malaria surveillance programme is predominantly dependent on passive surveillance carried out by different levels of the public health facilities. Monthly malaria cases are reported from the community level Sub-health Post (SHP) and Health Post (HP) to the Primary Health Care (PHC) centre, then to the District (Public) Health Office, and finally to the EDCD via the monthly health management information system (HMIS). Apart from the HMIS, there is a weekly early warning reporting system (EWARS) of admitted malaria cases and deaths from hospitals, a monthly global fund report, weekly community sentinel surveillance for outbreaks, a monthly logistics management report and an annual report to the EDCD. Malaria diagnosis includes microscopy in all public health facilities and pan-specific antigen RDT where microscopy is not available (some HPs and all SHPs) [6]. Malariometric indicator data at the national level were collected for the years between 1963 and 2012. National level data on malaria cases classified by origin (i.e. either indigenous or imported) were available only after 2001. In some years, a large number of malaria cases were unclassified by type of origin. For analysis, these were assigned to the indigenous and imported case categories based on the known proportion of those cases for the respective years. For example, if ten cases from a particular region were reported as unclassified, 30 as indigenous and 20 as imported, then the proportion of those ten unclassified cases of this year that were assumed to likely have been indigenous ones would be 10 × (30/(30 + 20) = 6. District level disaggregated data were available only from 2004 to 2012. Therefore, all high (13) and moderate risk (18) districts, which accommodate more than 90% of the total reported confirmed malaria cases of the country, were further analysed to compare the malariometic indicators between 2004 and 2012. An additional reason for choosing the data set of 2004–2012 for detailed analysis is that malaria intervention programmes, such as the distribution of ACT and LLINs, started in 2004 and 2005, respectively, with GFATM support [5,6]. All of the malaria cases used for analysis was confirmed cases (i.e. either by microscopy or RDT). Information on clinically suspected malaria cases was used only where explicitly stated. The classification of malaria cases was not complete between 2007 and 2009 with some districts reporting more than 50% unclassified cases by origin, sex and age groups. Therefore, only data that had more than 80% complete report from each district per year were included in the analysis. Number of LLINs distributed between 2005 and 2012, LLINs coverage and number of households protected by indoor residual spraying (IRS) were obtained from Nepal malaria report published by the EDCD. In addition, results of Nepal demographic and health survey 2006 and 2011 were reviewed to estimate percentages of households with at least one mosquito net.

In order to control for other factors that might influence the observed trends of malaria indicators, the following additional comparison indicators were used [10,11]: i) new outpatient visit per year for all cause consultation which was reported as percentage of total targeted population to observe changes in health-seeking behaviour and access to health facilities over the years and ii) incidences of childhood diarrhoeal diseases (CDD) and acute respiratory infection (ARI) from annual reports of the DoHS. This helps to observe trend in malaria indicators are not by other factors that also affect trends of other diseases in a similar way. An additional reason for choosing these indicators is that these diseases control programmes have similar community outreach like malaria and all are covered by the community based integrated management of childhood illness (CB-IMCI) programme since 1997 and were scaled up in phase wise manner covering all the districts of Nepal in the mid of 2010. The monthly rainfall in millimetres (mm) and air temperature (minimum, maximum and mean) in degree Celsius (°C) were obtained from seven meteorological stations of Department of Hydrology and Meteorology, Government of Nepal which are located within the study districts of lowlands terai to assess trends in climactic variables and control for the potential effect on malaria transmission.

### Data analysis

The data were entered in Microsoft Excel and analysed in the R computing software [12]. *Plasmodium vivax* is the predominant species that causes around 80-90% of the total malaria cases, but *Plasmodium falciparum* is the main cause of malaria outbreaks in Nepal [13]. Therefore, the trend of malaria incidence and the proportion of *P. falciparum* cases in Nepal (1963–2012) and the proportion of malaria cases by origin (2001–2012) were analysed. The incidence of malaria was estimated using the annual number of reported confirmed malaria cases as the numerator and the official number of population at risk of malaria estimated by EDCD as the denominator and expressed as per 10,000 population at risk. Similarly, the incidences of *P. vivax* and *P. falciparum* were calculated. Finally, a detailed comparison of the malaria indicator data of the years 2004 to 2012 was performed to examine the changes after the introduction of the GFATM programme, and to assess the feasibility for the country to embark towards elimination. Two methods were used to test the significance of improvement in malaria indicators over different periods taking 2004 as a reference period. Proportions and mean values with 95% confidence intervals (CI) on the estimates as population samples, and risk ratios (RR) were calculated to observe differences in proportions between discrete time periods [14]. First, the proportions of malaria parasite infections among sex and age groups were compared to test changes in the proportions between two discrete time periods 2004 and 2012 using a chi-square test. Second, log-linear regression assuming negative binomial distribution was used to test changes in malaria and non-malaria indicators over different periods compared to the reference period 2004 [11,14-16]. The negative binomial model (NBM) is an extension of Poisson model for incidence rates which allows for the over dispersion that commonly occurs for diseases count data, and is reported to be a robust analysis with respect to count data sets [15,17]. The log of the incidence rate is assumed to be linearly associated with interventions of different time periods, and the model parameters after exponentiation can be interpreted as RR which is similar to relative risk or relative incidence [16]. The 95% CI of RR which does not include one in the range was considered as being a significant change in the indicator. Linear regression was also employed to log transformed annual malaria incidence data of 31 malaria risk districts as well as of whole country to test the significance of decline over the periods 2004 to 2012 [14,18]. To detect autocorrelation, residuals of models were plotted against time and Durbin-Watson statistic was used to test for serial autocorrelation of the error terms in the regression which yielded values less than two indicating no evidence of serious autocorrelation [19]. In addition, the month-to-month trend in climatic variables between 2003 and 2012 were analysed using time series plots. These trends and findings were then compared with those described in the literature.

### Ethical considerations

The Ethical Review Board of the Nepal Health Research Council (NHRC) approved the conduct of this study. Only data that had been approved and documented by the EDCD, DoHS, Government of Nepal were used.

## Results

### Annual trends of confirmed malaria incidence and *Plasmodium falciparum* proportion in Nepal (1963 – 2012)

**Figure 2:**
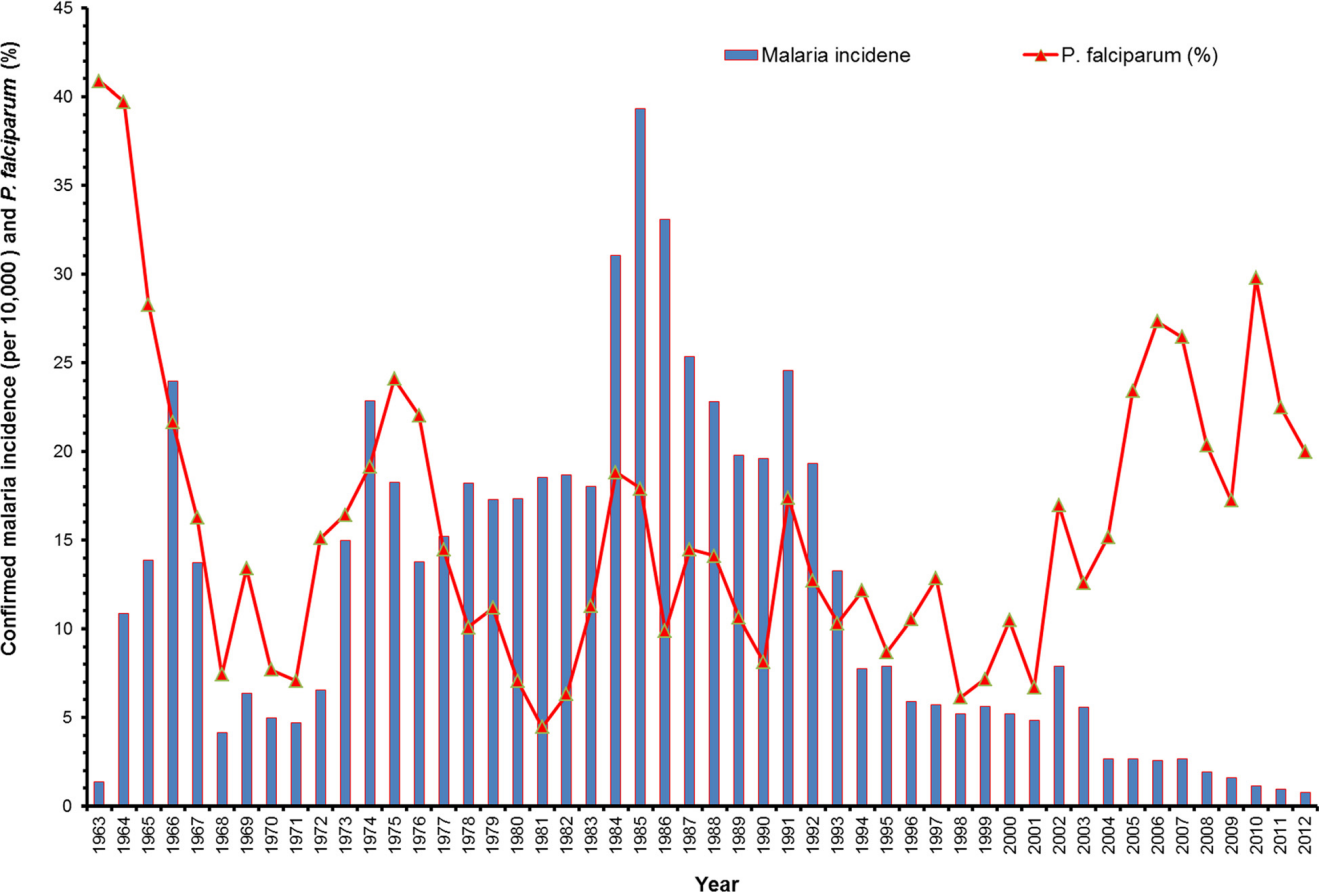
**Yearly trends of malaria incidence and *Plasmodium falciparum *** **malaria proportion in Nepal (1963–2012).**

The trend of confirmed malaria incidence and the proportion of *P. falciparum* cases over the period of 1963–2012 are shown in Figure 2. The proportion of *P. falciparum* cases was higher at the beginning of the 1960s (more than 40%), and cases were reduced to 2,500 by the end of the 1960s. The resurgence of malaria in Nepal started at the beginning of the 1970s, culminating in,14,647 cases in 1974 (19.1% of these *P. falciparum*). By 1976, this was reduced to 10,123 cases (22% *P. falciparum*). The number of malaria cases again started to rise in 1985 when they exceeded 42,321 (17.9% *P. falciparum*), the highest malaria caseload ever recorded in Nepal. However, the number of annual cases was reduced to around 22,000 by the end of the 1980s. Nepal again experienced a malaria outbreak at the beginning of the 1990s. The number of malaria cases rose to 29,000 (17.4% *P. falciparum*) in 1991. Malaria cases peaked again to 12,786 (17% *P. falciparum*) and 9,506 cases (12.9% *P. falciparum*) in 2002 and 2003, respectively. After this, annual case numbers were maintained below 5,000 in Nepal. Despite a steady decline in the number of malaria cases after 2003, the proportion of *P. falciparum* cases increased reaching 27.3% in 2006 and 29.8% of the total confirmed cases in 2010, the highest proportion of *P. falciparum* in Nepal ever recorded after the 1970s.

**Figure 3:**
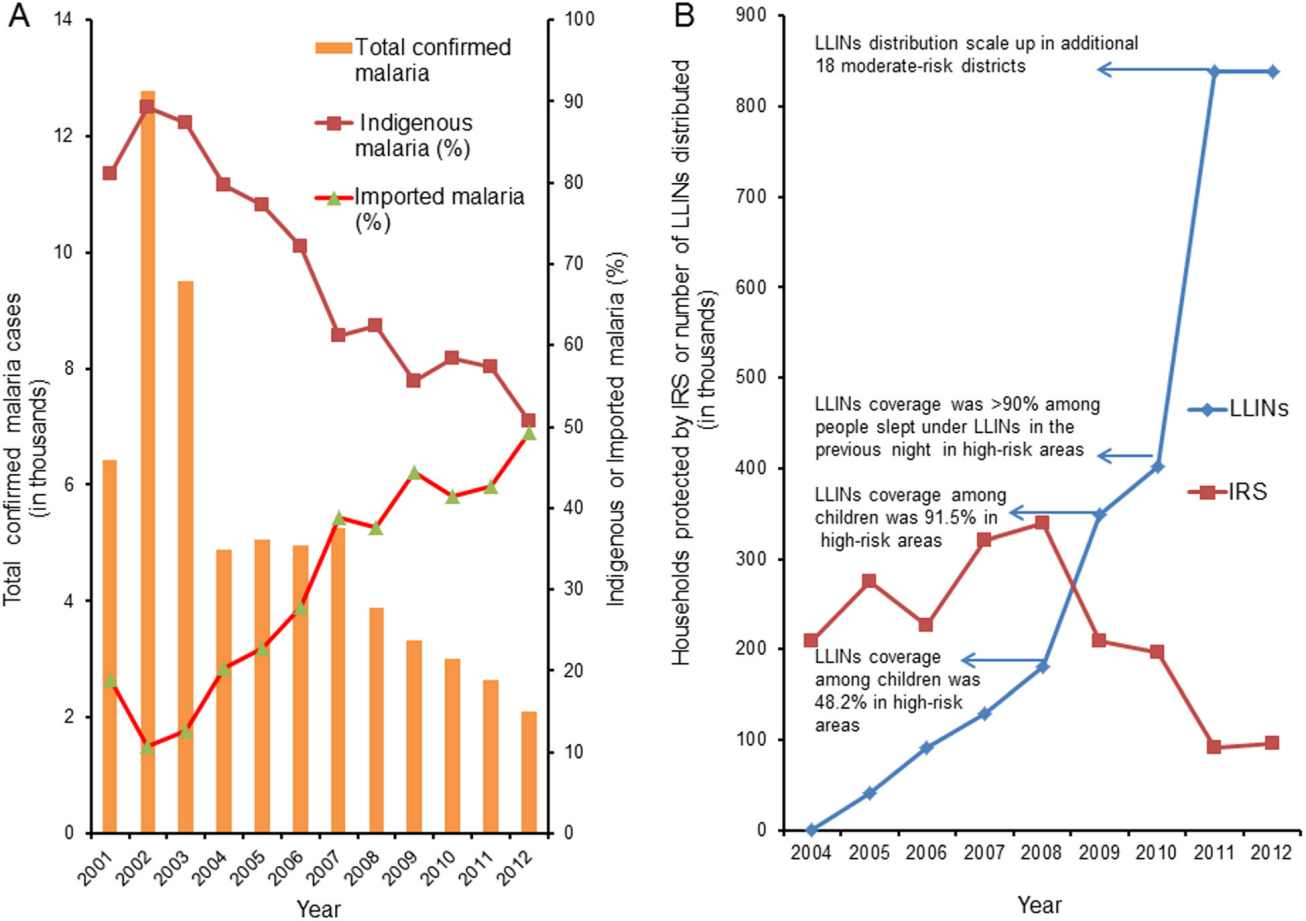
**Total confirmed malaria cases by origin and vector control interventions.** Panel **A** shows total confirmed malaria cases by origin between 2001 and 2012. Panel **B** shows households protected by indoor residual spraying (IRS) and long lasting insecticidal nets (LLINs) distributed between 2004 and 2012. In high-risk foci not covered by LLINs, IRS is done twice a year. The first round of IRS is undertaken during pre-monsoon (April-May) and the second round in monsoon (July-August) each year.

**Figure 4:**
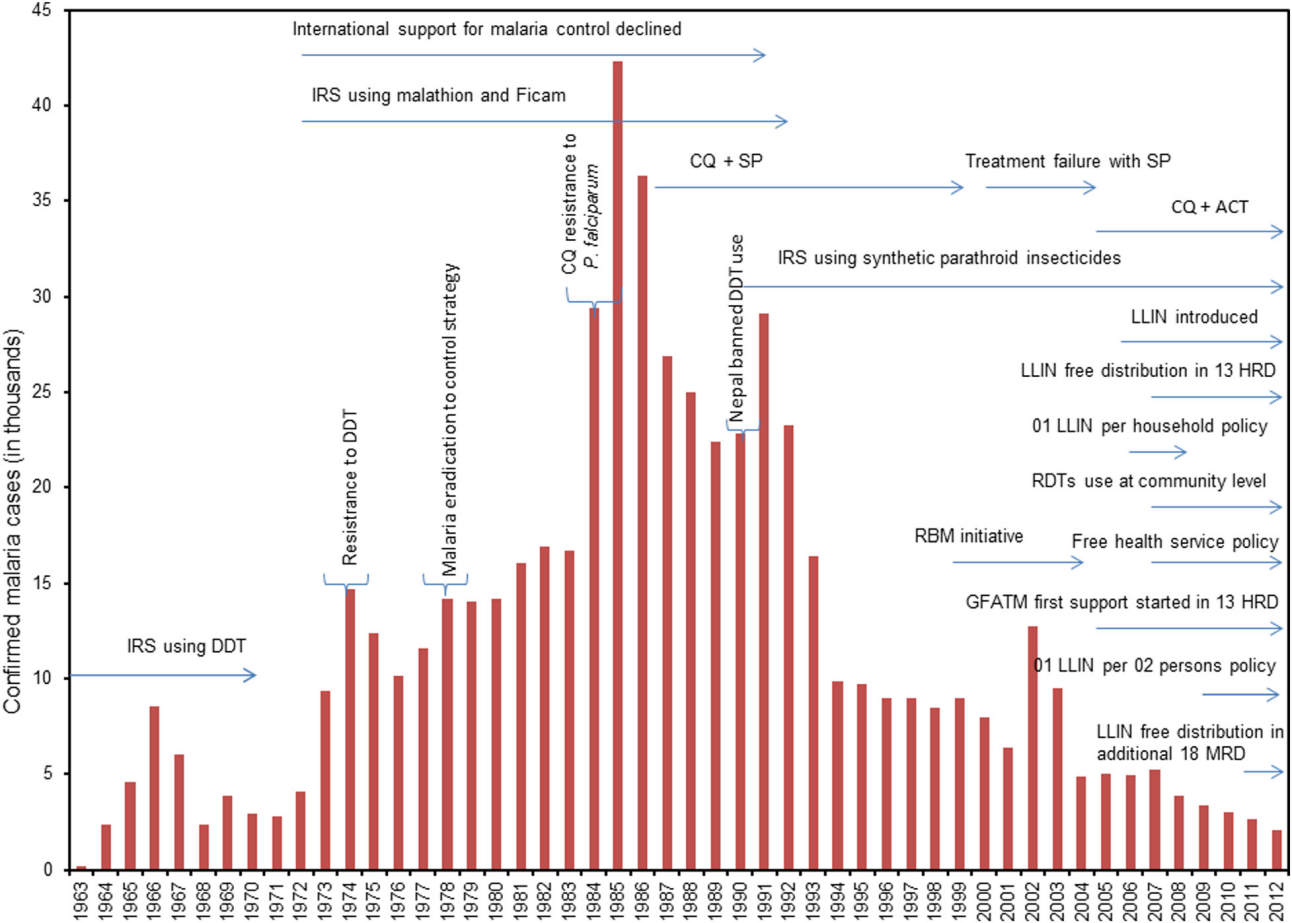
**Total confirmed malaria cases and important milestones on malaria control in Nepal (1963–2012).** During 2006–2008, long lasting insecticidal nets (LLINs) were distributed with policy of one LLIN per household in 13 high-risk districts (HRD). LLINs are being distributed at the rate of one LLIN per two persons in HRD and moderate-risk districts (MRD) since 2009 and 2011, respectively. Each bar with distinct peak values was attributed to malaria epidemics mainly caused by *P. falciparum* malaria. Treatment failure of *P. falciparum* malaria in Nepal has been shown for chloroquine (CQ) and sulfadoxine-pyrimethamine (SP) but clinical CQ resistance in *P. vivax* has not been reported in Nepal. Artemisinin combination therapy (ACT) was introduced for the treatment of uncomplicated *P. falciparum *in 2004.

Nepal has made significant progress in controlling malaria transmission over the past decade: The total number of confirmed malaria cases declined by 84% (from 12,750 in the year 2002 to 2,092 in 2012), the number of diagnosed *P. falciparum* cases by 81% (from 2,165 in 2002 to 418 in 2012), and only one malaria death was reported in 2012. However, both the proportions of *P. falciparum* (Figure 2) and imported malaria cases have been in an increasing trend after 2001 (Figure 3A). The proportion of imported malaria cases was less than 20% in 2002 but increased to 50% by 2012. The vector control intervention (i.e. LLINs distribution and households protected by IRS) is given in Figure 3B. The IRS is gradually replaced by LLINs coverage in malaria-risk districts. Total confirmed malaria cases and important milestones on malaria control in Nepal during the last 50 years are summarized in Figure 4.

### Changes in malariometric indicators of 31 malaria risk districts in Nepal from 2004 to 2012

**Figure 5:**
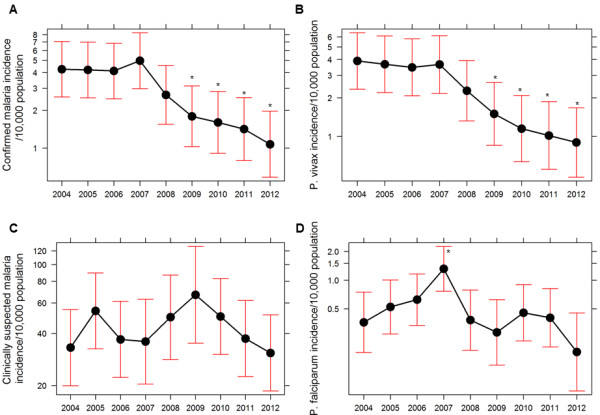
**Trends on malariometric indictors of 31 malaria risk districts in Nepal between 2004 and 2012.** Panel **A**, **B**, **C** and **D** show trends of confirmed malaria incidence, *Plasmodium vivax* malaria incidence, clinically suspected malaria incidence and *Plasmodium falciparum* malaria incidence, respectively. Graphs show trend plot, mean and 95%CI. The symbol (*) represents a statistically significant difference at *P* < 0.05 (Two-tailed).

The comparison of malariometric indicators of 31 malaria-risk districts for the years 2004 to 2012 showed a statistically significant improvement except few indicators. There were significant changes in proportions of malaria parasite infections among age and sex groups (Table 1). Males older than five years had higher infection rates with both *P. vivax* and *P. falciparum* malaria compared to females and to males under five years of age. Compared with 2004, the proportion of *P. vivax* has increased by15% among males above five years (RR = 1.15, 95% CI = 1.09-1.21) and has declined among females above five years by 47% (RR = 0.53, 95% CI = 0.4-0.6) in 2010. In contrast, the proportion of *P. falciparum* has increased in all age and sex groups with a statistically significant increment among children of both sex groups and males above five years. The incidence of confirmed malaria was static in 2005 and 2006 compared to 2004, and increased to peak in 2007 which coincides with RDT use at community level and the introduction of free health service policy to remove user fees in 2007. After achieving LLINs coverage in high-risk areas by more than 50% (Figure 3B), the confirmed malaria incidence declined in 2008 with a statistically significant decline starting from 2009 (Figure 5A). Compared with 2004, there was a decline by 58% in 2009 (RR = 0.42, 95% CI = 0.2-0.82), 63% in 2010 (RR = 0.37, 0.19-0.74), 73% in 2011 (RR = 0.27, 95% CI 0.19-0.74) and 75% in 2012 (RR = 0.25, 0.19-0.74). This decline is further supported by a significant decline in the incidence *P. vivax* malaria (Figure 5B). In contrast, compared with 2004, the incidences of *P. falciparum* and clinically suspected malaria have not declined significantly (Figure 5C and D). Furthermore, the analysis of linear regression on log transformed incidence of the confirmed malaria also showed a statistically significant decline by 18% per year in 31 malaria risk districts (95% CI, 12-24%) and 16% per year in the whole country (95% CI, 12-20%) (see Additional files 1 and 2), respectively.

**Table 1:** Comparison of malaria parasite infections by age and sex groups of high and moderate risk 31 districts in Nepal for the years 2004 and 2012

**Variables**	**Year 2004 N (%)**	**Year 2012 N (%)**	**Risk ratio (95% CI)**
*P. vivax*			
< 5 years male	44 (1.1)	28 (1.7)	1.46 (0.91-2.34)
< 5 years female	34 (0.9)	16 (1)	1.08 (0.60-1.96)
> 5 years male	2078 (54)	1037 (62.5)	1.15 (1.09-1.21)*
> 5 years female	1239 (32.3)	288 (17.3)	0.53 (0.48-0.6)*
*P. falciparum*			
< 5 years male	6 (0.2)	13 (0.8)	5 (1.9-13.13)*
< 5 years female	3 (0.1)	9 (0.5)	6.92 (1.87-25.53)*
> 5 years male	289 (7.5)	199 (12)	1.59 (1.33-1.88)*
> 5 years female	138 (3.6)	70 (4.2)	1.17 (0.88-1.55)
Total confirmed malaria	3831 (100)	1660 (100)	ND

ND means not determined.

*Significant difference at *P* < 0.001(Two-tailed).

### Changes in non-malaria indicators of 31 malaria risk districts in Nepal from 2004 to 2012

**Figure 6:**
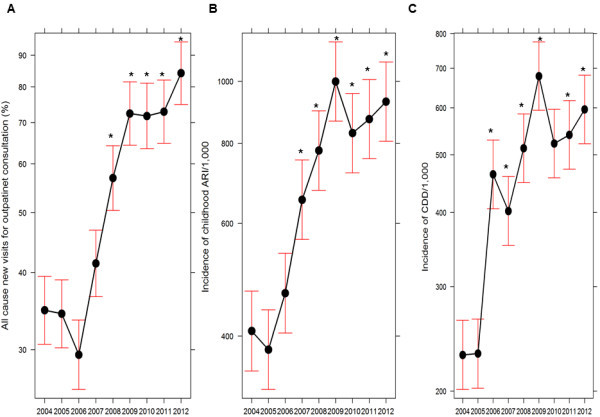
**Trends on non-malaria indicators of 31 malaria risk districts in Nepal between 2004 and 2012.** Panel **A**, **B** and **C** show trends of all cause new visits for outpatient consultation, incidence of acute respiratory infections (ARI) among children and incidence of childhood diarrhoeal diseases (CDD), respectively. All cause new visits for outpatient consultation was calculated as percentage of target population of each district. This includes a very small portion of malaria patients for outpatient consultation. Due to unavailability of complete data of outpatients for malaria consultation, only non-malaria consultation portion could not be computed. Graphs show trend plot, mean and 95% CI. The symbol (*) represents a statistically significant difference at *P* < 0.05 (Two-tailed). The incidences of ARI and CDD were computed per 1,000 children under five years (new visits or cases).

There was a significant increase in the proportion of outpatient visits for all cause consultation in health facilities over the years compared to the baseline year 2004 (Figure 6A). The incidences of ARI and CDD have also significantly increased over the years compared to 2004 (Figure 6B and C). Both malaria and non-malaria indicators generally followed similar trend from 2004 to 2007 but only malaria incidence followed significant declining trend from 2008 and non-malaria indicators followed statistically significant increasing trend.

### Effect of climatic variation on malaria transmission in 31 malaria risk districts in Nepal from 2004 to 2012

**Figure 7:**
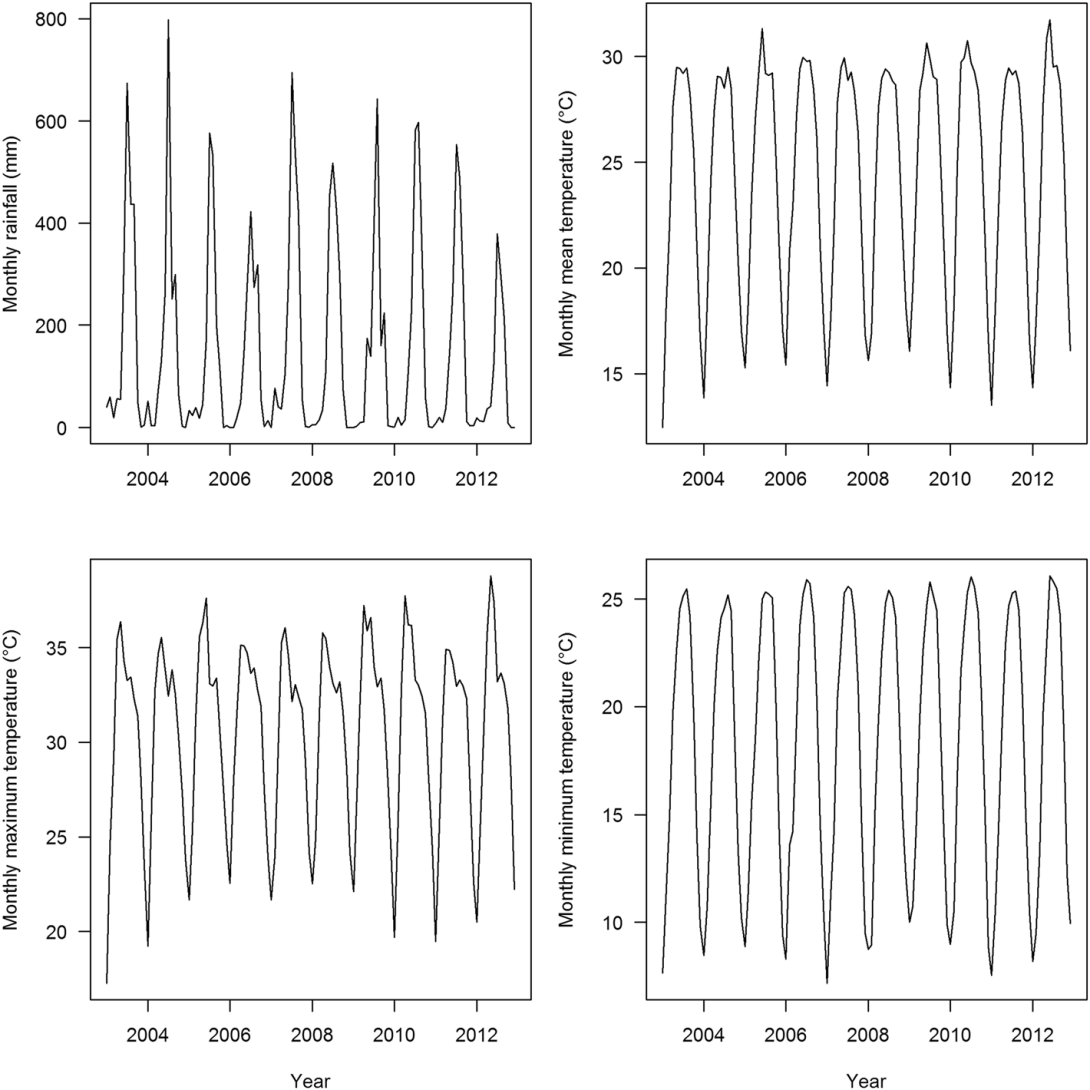
**Trends in monthly climatic variables between 2003 and 2012.** The monthly data of each climatic variable were taken from records of seven meteorological stations of lowlands terai districts which belong to high and moderate-risk malaria districts.

There were no noticeable anomalies or significant changes in rainfall and temperature trends between 2003 and 2012 (Figure 7) that could plausibly affect the observed trend of malaria transmission in lowland districts from where majority of the confirmed cases (>80%) were reported. Seasonality of both rainfall and temperature was normal during the study period.

## Discussion

The analysis of the available epidemiological data of malaria in Nepal between 1963 and 2012 shows a fluctuating trend of both confirmed malaria cases and the proportion of *P. falciparum.* The proportion of *P. falciparum* cases was higher at the beginning of the 1960s and drastically decreased after 1965. This may have been an effect of the national programme on malaria vector control by means of dichloro-diphenyl-trichloroethane (DDT) house-spraying which commenced in 1959 and had covered all parts of Nepal below 1,200 m above sea level by the year 1965 [20]. As a result of this effective nationwide anti-malaria programme, a large area of the lowlands of Nepal was practically malaria-free and the number of reported cases reduced to a minimum by the end of the 1960s [21,22]. The proportion of *P. falciparum* cases in Nepal reached a minimum of around 7% by 1971 [21,22]. At that time, the trends of the total malaria cases and the proportion of *P. falciparum* were similar to those in neighbouring India [23]. This has been related to reports stating that *Anopheles minimus* had been almost eliminated from Nepal by the control programme [5,20,22,24]. However, molecular tools were not available at that time and closely related or morphologically cryptic mosquito species were often misidentified. In particular, morphological characters of immature stages and adults of *A. minimus*, *Anopheles aconitus* and *Anopheles fluviatilis* are overlapping and polymorphic [25]. Thus, the alternative hypothesis cannot be refuted that the reported disappearance of *A. minimus* from Nepal at that time might have reflected taxonomic uncertainty or confusion with one or more other common *Anopheles* species, rather than selective elimination of *A. minimus*.

The resurgence of malaria in Nepal during the early 1970s was due to technical, financial, administrative, and logistic support problems coupled with the discovery that *Anopheles annularis* now functioned as a vector of malaria in the southern terai belt, leading to a massive epidemic in Parsa, Kapilbastu, Rupandehi, and Nawalparasi districts of the central and western terai [5,22,24,26]. Furthermore, during the 1970s and 1980s, international support for malaria control declined rapidly because of economic and financial crises causing malaria resurgence and epidemics in many countries, particularly on the Indian subcontinent [7]. Attempts to control the epidemic by IRS with DDT were not effective due to the development of resistance against DDT in *A. annularis*[5,24,26]. Nevertheless, during the late 1970s, rigorous efforts that involved changing the insecticide from DDT to the organo-phosphorous compound malathion, the bendiocarb-carbamate Ficam and larviciding with Abate were undertaken to control the epidemic in the western region of Nepal, and the number of cases was reduced to 10,123 by 1976 [5,24].

In July 1978, a review of the malaria programme’s objectives and strategy resulted in a transformation of the malaria eradication programme to malaria control according to WHO’s global strategy of malaria control [5,24,27]. The decade of the 1980s was a setback for the malaria control programme in Nepal. The highest number of malaria cases in 1985 was due to a large-scale epidemic in the far-western region [5,24,28,29]. The number of annual cases was reduced to around 22,000 by the end of the 1980s with concerted efforts such as changing the anti-malarial drug policy from chloroquine (CQ) to sulphadoxine-pyrimethamine (SP) against *P. falciparum* and changing insecticides, establishing passive case detection volunteer posts in epidemic and epidemic-prone areas, and the establishment of free drug distribution centres [5,24,27].

A high number of malaria cases at the beginning of the 1990s was attributed to another malaria epidemic in the central and far-western regions of Nepal. Case numbers were brought down to below 10,000 by 1995 with intensive efforts that included mosquito control by continued IRS in epidemic areas using the synthetic pyrethroid lambda-cyhalothrin [5,24]. Beside this, the country had to manage 1,600 (51% *P. falciparum*) and 1,461 (43% *P. falciparum*) additional malaria cases in 1994 and 1995, respectively, due to an influx of refugees from Bhutan into eastern Nepal [5,24]. However, these refugee data between 1994 and 2000 are not included in the present analysis.

The peak values of malaria positive cases in 2002 and 2003 were contributed by an outbreak of malaria in Krishanpur, Jhallari and Daizi village development committees (VDC) of Kanchanpur district in far-western Nepal [30,31]. The cause of the outbreaks in Kanchanpur and Nawalparasi districts was treatment failure of *P. falciparum* cases with SP [32-34]. In line with these findings, artemether-lumefantrine (AL, Coartem®) was introduced against uncomplicated *P. falciparum* malaria in 2004 in high-priority, malaria-endemic districts of Nepal, and throughout the country since 2009 [13]. The increase in the proportion of *P. falciparum* cases after 2004 was mainly due to increasing numbers of *P. falciparum* cases in Jhapa district in 2005 and an outbreak of *P. falciparum* malaria in Banke district in 2006 [5] which claimed 36 deaths [13]. The malaria outbreak in Banke district occurred in September/October 2006 after heavy flooding and prolonged heavy rainfall during the preceding monsoon season. Entomological investigations in affected villages revealed a high density of *A. fluviatilis* and a low density of *A. annularis*[5,24]. Previously, Banke district had been classified as a low-risk district and no vector control measures had been in place over the previous ten to 15 years. This example shows that an outbreak of malaria, especially of *P. falciparum* malaria, may occur at any time even in low-risk areas following changes in the ecology, vector dynamics or extreme weather events such as heavy rainfall and flooding.

The highest proportion of *P. falciparum* cases in 2010, reaching 29.8% of the total confirmed malaria cases without any outbreak of malaria in the country, is attributed to a large number of imported cases (1,314 of total 3,004 cases). The drastic decline in indigenous malaria cases but continuous introduction of imported cases has increased the proportion of both imported and *P. falciparum* malaria cases in Nepal in the last decade.

Nepal does appear to have a considerable proportion of mixed infections of *P. falciparum* and *P. vivax*, but only limited evidence is available in the absence of molecular studies [35]. In the present analysis of total confirmed malaria cases, only about 1% had been diagnosed as mixed (*P. vivax* and *P. falciparum*) infections. Due to their low number, these mixed infections are usually reported as *P. falciparum* cases in Nepal. In one study, PCR analysis showed that there were no *Plasmodium malariae* or *Plasmodium ovale*, but *P. falciparum* and *P. vivax* mixed infections constituted 17% of the total sample (113 cases) [36]. Another study found no mixed infection by microscopy, but 5.1% (five of 98 samples) were diagnosed as mixed (*P. vivax* and *P. falciparum*) infections by PCR [37]. Thus, the apparently low percentage of mixed infection in the data of the present study may be an artefact of testing malaria positive cases only by microscopy and RDTs. A recent study from two districts of far-western Nepal shows a 17% relapse rate in *P. vivax* malaria (23 of 137 cases) with a high proportion of males from the age group 11–20 years [38]. Although this relapse rate is not known at the national level, a high relapse rate may be a hurdle to achieving the malaria elimination goal.

The annual blood examination rate of Nepal is below 1%. Not all of the collected blood slides were tested for malaria parasites due to various reasons including lack of human resources and laboratory facilities [3,24]. Therefore, the observed increasing number of clinically suspected malaria cases and the corresponding decline in confirmed malaria cases in Nepal may be attributed to this diagnostic gap.

The seasonal variation of malaria case numbers in Nepal shows a distinct peak in either the monsoon or post-monsoon season. The peak densities of the primary malaria vector in Nepal, *A. fluviatilis*, which was shown to be responsible for malaria transmission up to 1,300 m altitude in Nepal, occur during and after the monsoon (July-December) in mountain valleys, before and after the monsoon (March-April and October-December) in the inner terai and following the monsoon (November-February) in the forested terai [5,24,27]. Most of the documented malaria outbreaks in Nepal were due to transmission by this species [5,24].

Malaria transmission is mainly confined to the southern districts of Nepal bordering India. Hence, a cross-border malaria strategy with India is very important. With this goal, the USAID Bureau for Asia and Near East (ANE) and USAID Nepal, in collaboration with the WHO, supported a regional initiative for Bangladesh, Bhutan, India and Nepal (BBIN) in the year 2000 to implement cross-border activities for the control of three vector-borne diseases (VBDs): malaria, leishmaniasis and Japanese encephalitis [39-41]. Despite these efforts, the issue of imported malaria cases in Nepal is not under control yet. This may be due to the fact that the proportion of *P. falciparum* cases is also in an increasing trend in the neighbouring country India although the number of malaria positive cases has been declining over the years [23]. Hence, large-scale migration across the southern border of Nepal with India and continued introduction of malaria cases among adult males could be seen as a major threat favouring possible focal outbreaks of *P. falciparum* malaria and hindering Nepal’s efforts towards malaria elimination [6,42]. The majority of imported cases had a history of travel to malaria endemic areas of India and predominantly were adult male labourers [6]. The total number of imported cases is dominated by *P. vivax*. However, among the total *P. falciparum* cases, the proportion of imported *P. falciparum* malaria is higher than that of indigenous *P. falciparum* malaria. The peak season of imported malaria cases in Nepal is the post-monsoon season during which migrating workers usually return home to celebrate major festivals in Nepal. The other countries of Southeast Asia that are in a pre-elimination phase (Bhutan and Sri Lanka) have experienced problems achieving elimination goals due to imported malaria [39,43,44], and Iran in 2011 moved backwards from elimination to pre-elimination phases in malaria control because of imported cases [45].

The total number of confirmed malaria cases in Nepal has drastically declined over that last decade. Thereby, Nepal has achieved and exceeded the malaria target of the Millennium Development Goals (MDGs) and universal coverage of malaria control interventions, and the Roll Back Malaria (RBM) targets of 2010. Contributing factors behind the static decline in the number of malaria positive cases may be the introduction of ACT for the treatment of uncomplicated *P. falciparum* cases since 2004 and the distribution of LLINs in high-risk priority districts since 2005 with support from the GFATM along with the delivery of free primary health care services from public health institutions since 2007 [2,4,5,13,24,46,47]. However, case numbers also declined in areas where GFATM intervention programmes were not in place. Although the reasons for this are not known, other factors, both human and climatic, may have played a role in reducing the malaria incidence [48]. For example, the percentage of households possessing mosquito nets in Nepal had increased from 61.3% in 2006 to 67.8% in 2011 [49,50].

A minimum requirement for malaria elimination is that the reproductive number under control (Rc) should be less than one [51]. The ratio of indigenous to imported cases can be taken as approximately indicative of the current value of Rc [52]. However, estimating Rc in Nepal is difficult due to unavailability of information on asymptomatic infections which also contribute to the reproduction of the infection, and because only a passive surveillance system exists for case detection with an annual blood examination rate accounting below 1%. Population prevalence surveys conducted in two high-risk malaria districts (Jhapa and Kanchanpur) in 2008 showed parasitic prevalence rates of 0.82% and 1.92%, respectively [6]. In the same year, the slide positive rates from the passive surveillance system in Jhapa and Kanchanpur were 4.7% and 1.6%, respectively, indicating that different results may be obtained according to the surveillance system used and the prevalence of asymptomatic malaria infections is still high at the population level.

The comparison of the incidence of confirmed malaria and *P. vivax* malaria of 31 malaria-risk districts in Nepal for the years 2004 to 2012 shows statistically significant decline following scale up of LLINs in high-risk areas and availability of ACT as first-line treatment to uncomplicated *P. falciparum.* The trend of the confirmed malaria observed in the present study is consistent with findings of a previous study [15].

All cause outpatient consultation visits (reported as percentage of new cases of total population) has increased significantly over the years which was similar to malaria incidences before 2008 (Figure 6A). Similarly, compared with 2004, incidences of ARI and CDD have significantly increased since 2007 and 2006, respectively (Figure 6B and C). These significant increasing trends of non-malaria indicators may be attributed to the introduction of free health service policy by the Government of Nepal in 2007 and scaled up of the CB-IMCI programmes in all districts of Nepal by 2010 [3].

No significant changes in rainfall and temperature pattern were identified that could explain the observed trend in malaria incidence of 31 malaria-risk districts which are mostly located in lowlands terai districts (Figure 7). The non-significant trend in temperature and rainfall in lowlands terai is consistent with findings of previous studies [53-55]. In contrast, the risk of malaria transmission in the temperate regions of Nepal is speculated to gradually increase because of climate change since global warming has more pronounced effects in the higher altitudes of Nepal compared to the lowlands of the terai [8,53,55-58]. A significant positive correlation between climate variables: rainfall and temperature, and number of confirmed malaria cases is reported in a study conducted in the high-risk Jhapa district [59]. Several recent studies show the increasing trend of epidemic potential of malaria in temperate regions and tropical highlands in different climate change scenarios [60-63]. Accordingly, the temperate region of Nepal is at risk of malaria due to climate change coupled with the expansion of infrastructure development such as road construction, hydropower development and the construction of ponds for fisheries. The distribution of the disease, which was previously believed to be confined to the forest and forest fringe regions of the terai lowlands and so-called inner terai valleys, is now observed to extend to an altitude of almost 2,000 m above sea level in the Himalayas of Nepal [3,15,64]. However, the effect of climate change on the epidemiology of malaria in Nepal has remained elusive in the absence of specific studies [64]. Hence, expansion of malaria in high altitude districts previously considered non-endemic for malaria transmission despite of significant decline of malaria incidence in lowlands terai and hill districts can be one of the challenges for achieving the malaria elimination goal.

The coverage of LLINs in malaria high-risk areas is reported more than 90% in 2010 and percentage of children sleeping under LLIN (last night) rose from 48.2% in 2006 to 91.5% in 2009 (Figure 3B) [6]. In Nepal, IRS has been a well-established vector control intervention for a long period. Therefore, the observed decline trend of malaria incidence may not be attributed to IRS as IRS activities were occurred throughout both pre-and intervention periods. Although unmeasured factors may have attributed to this significant decline of confirmed malaria and *P. vivax* malaria, available health facilities data indicate that this significant decline coincided with the scale up of free LLINs distribution policy of one LLIN per two persons in high-risk areas of 31 districts and AL as the first-line of treatment for uncomplicated confirmed *P. falciparum* malaria throughout the country since 2009 [6,13].

Despite a statistically significant decline in the incidence of total confirmed and *P. vivax* malaria, there is no significant decline in *P. falciparum* and clinically suspected malaria incidences. The significant increase in proportion of *P. vivax* and *P. falciparum* among above five years age group is mainly contributed by imported malaria cases among male labourers. Surprisingly, both total number as well as proportion of *P. falciparum* has increased among children in 2012 compared to 2004 which may be because of two reasons. First, increased case detection rate and reporting of malaria among children in 2012 compared to in 2004 which is consistent with increasing trend of outpatient consultation, CDD and ARI over the years (Figure 6) along with the scale up of the CB-IMCI programme in Nepal covering all districts by mid of 2010. Second, imported cases of *P. falciparum* among children (e.g. out of total 21 reported *P. falciparum* cases among children in 2012, seven were imported cases). These facts show that malaria, especially *P. falciparum* malaria, persists in high-risk districts despite massive interventions such as LLINs and IRS in these districts. This may be due to the fact that such interventions alone cannot easily break the transmission cycle of *P. falciparum* malaria [65]. The resurgence of *P. falciparum* malaria immediately after decrease in insecticidal net coverage is reported in Rwanda [11]. Moreover, the resistance of malaria vectors to pyrethroids, the possibly short-lived efficacy of LLINs (e.g. in Zanzibar [66]) and *P. falciparum* resistance development to artemisinin derivatives (in Cambodia [67,68]) challenge malaria control and elimination activities worldwide.

The magnitudes of declines found in malaria indicators are in line with those reported from similar studies in neighbouring countries: Bhutan, India and China [39,69-71]. Although attempt is made to include completely reported malaria indicator data, the findings of this study should be interpreted with a caution as it is entirely dependent on retrospective surveillance data. These results may represent actual time trends in malaria incidence at health facilities which may not represent trends of true malaria incidence at the population level [14]. However, this study provides important information about past milestones, the present malaria situation and challenges on the path towards malaria elimination in Nepal.

## Conclusions

The 50-year malaria control experience of Nepal shows that the malaria resurgence and major outbreaks in the past were caused or aggravated by resistance development against anti-malarial drugs and insecticides, and by economic and financial crises affecting international support for malaria control. Despite these challenges, Nepal has made significant progress in the past decade, achieving and exceeding the malaria target of the MDGs, universal coverage of malaria control interventions and the RBM targets of 2010. A series of interventions are likely to have contributed to this decline. These include a drug policy change from monotherapy to AL, IRS in high-endemic foci, the distribution of LLINs in high-endemic areas, and enabling factors such as economic development and free health service delivery from government health institutions. Based on the evaluation of its national malaria control programme in 2010, Nepal has adopted a long-term malaria elimination strategy 2011–2026 with the vision of a malaria-free Nepal by 2026. However, considerable challenges lie ahead. These range from laboratory confirmation of clinically suspected malaria cases and their treatment, drug resistance especially of *P. falciparum*, relapse/re-infection with *P. vivax*, and vector control in the face of increasing insecticide resistance, to climate change and managing the large numbers of imported cases from foreign countries and in Indian border districts. In order to achieve the ambitious malaria elimination goal, more operational research is needed to generate local evidence on the sustainability and risks of malaria elimination efforts in Nepal. The regional initiative for the BBIN should be strengthened to implement cross-border activities for the control of VBDs including malaria. Border malaria check posts need to be established and all fever cases should be screened at the border crossing check posts. More importantly, high risk groups who are currently not covered by the IRS/LLIN strategy (e.g. populations affected by natural disasters, labourers returning from malaria endemic areas, forest dwelling populations) should be provided protection from malaria vectors. Furthermore, the current preventive and control measures should be strengthened to sustain and consolidate the achievements made so far with improved community involvement without reducing national and international support.

## Competing interests

The authors declare that they have no competing interests.

## Authors’ contributions

MD designed the study, collected and analysed the data, and wrote the manuscript. BA participated in revision of the manuscript and data analysis. UK conceived the study and reviewed the draft manuscript. All authors read and approved the final manuscript.

## Supplementary Material

Additional file 1 **Linear trend of confirmed malaria incidence in 31 malaria risk districts (2004–2012).** The linear regression of confirmed malaria incidence shows significant decline between 2004 and 2012 in 31 malaria-risk districts which accommodate more than 90% reported confirmed cases each year. Click here for file

Additional file 2 **Linear trend of confirmed malaria incidence in Nepal (2004–2012).** The linear regression of confirmed malaria incidence shows significant decline between 2004 and 2012 in Nepal. Click here for file

## References

[B1] WHO World Malaria Report 2013 2013 Geneva: World Health Organization

[B2] WHO World Malaria Report 2011 2011 Geneva: World Health Organization

[B3] DoHS Annual Report 2010/2011 2012 Kathmandu: Department of Health Services, Ministry of Health and Population, Government of Nepal

[B4] EDCD Nepal Malaria Strategic Plan 2011–2016 (Revised Version- December 2011) 2011 Kathmandu: Epidemiology and Disease Control Division, Department of Health Services, Ministry of Health and Population, Government of Nepal

[B5] EDCD The Internal Assessment of Malaria and Kala-azar Control Activities 2004, 2005 and 2006 2007 Kathmandu: Epidemiology and Disease Control Division, Department of Health Services, Ministry of Health and Population, Government of Nepal

[B6] EDCD Nepal Malaria Report 2010–2012 2013 Kathmandu: Epidemiology and Disease Control Division, Department of Health Services, Ministry of Health and Population, Government of Nepal

[B7] Najera JA Gonzalez-Silva M Alonso PL Some lessons for the future from the Global Malaria Eradication Programme (1955–1969) PLoS Med 2011 8 e1000412 10.1371/journal.pmed.1000412 21311585 PMC3026700

[B8] MoE National Adaptation Program of Action to Climate Change (NAPA) 2010 Kathmandu: Ministry of Environment, Government of Nepal

[B9] CBS National Population and Housing Census 2011. Vol. 1 2012 Kathmandu: Central Bureau of Statistics, National Planning Commission Secretariat, Government of Nepal

[B10] Otten M Aregawi M Were W Karema C Medin A Bekele W Jima D Gausi K Komatsu R Korenromp E Low-Beer D Grabowsky M Initial evidence of reduction of malaria cases and deaths in Rwanda and Ethiopia due to rapid scale-up of malaria prevention and treatment Malar J 2009 8 14 10.1186/1475-2875-8-14 19144183 PMC2653503

[B11] Karema C Aregawi MW Rukundo A Kabayiza A Mulindahabi M Fall IS Gausi K Williams RO Lynch M Cibulskis R Fidele N Nyemazi J Ngamije D Umulisa I Newman R Binagwaho A Trends in malaria cases, hospital admissions and deaths following scale-up of anti-malarial interventions, 2000–2010 Rwanda Malar J 2012 11 236 10.1186/1475-2875-11-236 PMC3502144 22823945

[B12] R Core Development Team R: A Language and Environment for Statistical Computing 2.15.2 2012 Vienna: R Foundation for Statistical Computing

[B13] EDCD National Malaria Treatment Protocol 2009 Kathmandu: Epidemiology and Disease Control Division, Department of Health Services, Ministry of Health and Population, Government of Nepal

[B14] Ceesay SJ Casals-Pascual C Erskine J Anya SE Duah NO Fulford AJ Sesay SS Abubakar I Dunyo S Sey O Palmer A Fofana M Corrah T Bojang KA Whittle HC Greenwood BM Conway DJ Changes in malaria indices between 1999 and 2007 in the Gambia: a retrospective analysis Lancet 2008 372 1545 1554 10.1016/S0140-6736(08)61654-2 18984187 PMC2607025

[B15] Kakchapati S Ardkaew J Modeling of malaria incidence in Nepal JRHS 2011 11 7 13 22911941

[B16] Nelson KE Galai N Safaeian M Strathdee SA Celentano DD Vlahov D Temporal trends in the incidence of human immunodeficiency virus infection and risk behavior among injection drug users in Baltimore, Maryland, 1988–1998 Am J Epidemiol 2002 156 641 653 10.1093/aje/kwf086 12244033

[B17] O’Hara RB Kotze DJ Do not log-transform count data Methods Ecol Evol 2010 1 118 122 10.1111/j.2041-210X.2010.00021.x

[B18] Behrens RH Carroll B Smith V Alexander N Declining incidence of malaria imported into the UK from West Africa Malar J 2008 7 235 10.1186/1475-2875-7-235 19000299 PMC2613412

[B19] Wagner AK Soumerai SB Zhang F Ross-Degnan D Segmented regression analysis of interrupted time series studies in medication use research J Clin Pharm Ther 2002 27 299 309 10.1046/j.1365-2710.2002.00430.x 12174032

[B20] Parajuli MB Shrestha SL Vaidya RG White GB Nation-wide disappearance of *Anopheles minimus* Theobald, 1901, previously the principal malaria vector in Nepal Trans R Soc Trop Med Hyg 1981 75 603 603

[B21] Sakya GM Present status of malaria in Nepal J Nep Med Ass 1981 19 21 28

[B22] Shrestha SL Dynamics of malaria transmission with reference to development projects in Nepal J Commun Dis 1985 17 287 292 3836251

[B23] Sharma VP Continuing challenge of malaria in India Curr Sci 2012 102 678 682

[B24] EDCD Internal Assessment of Malaria Control and Kala-azar Elimination Activities 2007, 2008 and 2009 2010 Kathmandu: Epidemiology and Disease Control Division, Department of Health Services, Ministry of Health and Population, Government of Nepal

[B25] Garros C Van Bortel W Trung HD Coosemans M Manguin S Review of the *minimus* complex of *Anopheles*, main malaria vector in Southeast Asia: from taxonomic issues to vector control strategies Trop Med Int Health 2006 11 102 114 10.1111/j.1365-3156.2005.01536.x 16398761

[B26] Shrestha SL Parajuli MB Reappearance of malaria in terai area of Nepal and incrimination of *A. annularis* van der Wulp J Nepal Med Assoc 1980 1 11 18

[B27] Rana KJ History of Malaria and Malaria Control in Nepal 2001 New Delhi: Aravail Printers and Publishers P.Ltd

[B28] Reisen WK Pradhan SP Shrestha JP Shrestha SL Vaidya RG Shrestha JD Anopheline mosquito (Diptera: Culicidae) ecology in relation to malaria transmission in the inner and outer terai of Nepal, 1987–1989 J Med Entomol 1993 30 664 682 8360891 10.1093/jmedent/30.4.664

[B29] Shrestha SL Pradhan S Shrestha JPB Shrestha JD Rajbhamdari Y Shrestha GL Swar TB Nushin MK Reisen WK Observations on anophelines and malaria ecology in the Far Western Region of Nepal, 1986 Bull Soc Vector Ecol 1988 13 332 342

[B30] DoHS Annual Report 2002/2003 2004 Kathmandu: Department of Health Services, Ministry of Health and Population, Government of Nepal

[B31] DoHS Annual Report 2003/2004 2005 Kathmandu: Department of Health Services, Ministry of Health and Population, Government of Nepal

[B32] Chand PB Wijeyaratne PM Pandey S Ansari MA Valecha N Assessment of therapeutic efficacy of anti-malarial drug against uncomplicated *Plasmodium falciparum* malaria in the Indo-Nepal border Jhapa district (Nepal) & Darjeeling district, West Bengal (India) J Nepal Health Res Counc 2003 1 57 59

[B33] Mittal PK Wijeyaratne PM Pandey S Status of insecticide resistance of malaria, kala-azar and japanese encephalitis vectors in Bangladesh, Bhutan, India and Nepal (BBIN) Env Health Project Activity Rep 2004 129 1 98

[B34] Wijeyaratne PM Valecha N Joshi AB Singh D Pandey S An inventory on malaria drug resistance in Bangladesh, Bhutan, India and Nepal Env Health Project Activity Rep 2005 130 1 43

[B35] Banjara MR Sirawaraporn W Petmitr S Imwong M Joshi AB Chavalitshewinkoon-Petmitr P Characteristics and risk factors of *Plasmodium falciparum* malaria in Eastern and Central Nepal Kathmandu Univ Med J 2009 7 378 382 10.3126/kumj.v7i4.2758 20502078

[B36] Ranjitkar S Schousboe ML Thomsen TT Adhikari M Kapel CM Bygbjerg IC Alifrangis M Prevalence of molecular markers of anti-malarial drug resistance in *Plasmodium vivax* and *Plasmodium falciparum* in two districts of Nepal Malar J 2011 10 75 10.1186/1475-2875-10-75 21457533 PMC3080351

[B37] Thapa S Hollander J Linehan M Cox-Singh J Bista MB Thakur GD Davis WA Davis TM Comparison of artemether-lumefantrine with sulfadoxine-pyrimethamine for the treatment of uncomplicated *falciparum* malaria in eastern Nepal Am J Trop Med Hyg 2007 77 423 430 17827354

[B38] Manandhar S Bhusal CL Ghimire U Singh SP Karmacharya DB Dixit SM A study on relapse/re-infection rate of Plasmodium vivax malaria and identification of the predominant genotypes of P. vivax in two endemic districts of Nepal Malar J 2013 12 324 10.1186/1475-2875-12-324 24041296 PMC3848640

[B39] Yangzom T Gueye CS Namgay R Galappaththy GN Thimasarn K Gosling R Murugasampillay S Dev V Malaria control in Bhutan: case study of a country embarking on elimination Malar J 2012 11 9 10.1186/1475-2875-11-9 22230355 PMC3278342

[B40] Wijeyaratne P Cross-border collaboration on vector-borne disease control in Bangladesh, Bhutan, India and Nepal J Nepal Health Res Counc 2002 1 32 42

[B41] Brantly E Wijeyaratne P Singh D Pandey S Intercountry Collaboration for Improving Surveillance and Control of Vector-borne Diseases Env Health Project Activity Rep 2004 136 1 38

[B42] WHO/SEARO Nepal Malaria Programme Review (7–16 June, 2010) 2011 New Delhi: World Health Organization Regional Office for South-East Asia

[B43] Abeyasinghe RR Galappaththy GN Smith Gueye C Kahn JG Feachem RG Malaria control and elimination in Sri Lanka: documenting progress and success factors in a conflict setting PLoS ONE 2012 7 e43162 10.1371/journal.pone.0043162 22952642 PMC3430652

[B44] Wickramage K Galappaththy GN Malaria burden in irregular migrants returning to Sri Lanka from human smuggling operations in West Africa and implications for a country reaching malaria elimination Trans R Soc Trop Med Hyg 2013 107 337 340 10.1093/trstmh/trt009 23584376

[B45] Galappaththy GN Fernando SD Abeyasinghe RR Imported malaria: a possible threat to the elimination of malaria from Sri Lanka? Trop Med Int Health 2013 18 761 768 10.1111/tmi.12097 23506152

[B46] EDCD National Malaria Control Strategic Plan: Nepal (2007/2008-2011/012) 2007 Kathmandu: Epidemiology and Disease Control Division, Department of Health Services, Ministry of Health and Population, Government of Nepal

[B47] WHO World Malaria Report 2012 2012 Geneva: World Health Organization

[B48] Hommel M Towards a research agenda for global malaria elimination Malar J 2008 7 1 S1 10.1186/1475-2875-7-1 19091033 PMC2604876

[B49] MoHP [Nepal], New Era and Macro International Inc Demographic and Health Survey 2006 2007 Kathmandu: Ministry of Health and Population [Nepal], New Era and Macro International Inc

[B50] MoHP [Nepal], New Era and ICF International Inc Demographic and Health Survey 2011 2012 Kathmandu: Ministry of Health and Population [Nepal], New Era and ICF International Inc, Carvelton, Maryland

[B51] Farrington CP Kanaan MN Gay NJ Branching process models for surveillance of infectious diseases controlled by mass vaccination Biostatistics 2003 4 279 295 10.1093/biostatistics/4.2.279 12925522

[B52] Cohen JM Moonen B Snow RW Smith DL How absolute is zero? An evaluation of historical and current definitions of malaria elimination Malar J 2010 9 213 10.1186/1475-2875-9-213 20649972 PMC2983111

[B53] Shrestha AB Wake CP Mayewski PA Dibb JE Maximum temperature trends in the Himalaya and its vicinity: an analysis based on temperature records from Nepal for the period 1971–94 J Clim 1999 12 2775 2786 10.1175/1520-0442(1999)012<2775:MTTITH>2.0.CO;2

[B54] Shrestha AB Wake CP Dibb JE Mayewski PA Precipitation fluctuations in the Nepal Himalaya and its vicinity and relationship with some large scale climatological parameters Int J Climatolol 2000 20 317 327 10.1002/(SICI)1097-0088(20000315)20:3<317::AID-JOC476>3.0.CO;2-G

[B55] Shrestha AB Aryal R Climate change in Nepal and its impact on Himalayan glaciers Reg Environ Change 2011 11 S65 S77 10.1007/s10113-010-0174-9

[B56] Practical Action Nepal Temporal and Spatial Variability of Climate Change over (1976–2005) 2009 Kathmandu: Practical Action Nepal

[B57] NCVST Vulnerability Through the Eyes of Vulnerable: Climate Change Induced Uncertainties and Nepal’s Development Predicaments 2009 Kathmandu: Institute for Social and Environmental Transition-Nepal (ISET-N, Kathmandu) and Institute for Social and Environmental Transition (ISET, Boulder, Colorado) for Nepal Climate Vulnerability Study Team (NCVST)

[B58] MoPE [Nepal] and UNEP Nepal Initial National Communication to the Conference of the Parties of the United Nations Framework Convention on Climate Change 2004 Kathmandu: Ministry of Population and Environment, Government of Nepal and United Nations Environment Programme

[B59] Bhandari GP Dhimal M Gurung S Bhusal CL Climate change and malaria in Jhapa district of Nepal: emerging evidences from Nepal J Health Manag 2013 15 141 150 10.1177/0972063413486026

[B60] Siraj AS Santos-Vega M Bouma MJ Yadeta D Ruiz Carrascal D Pascual M Altitudinal changes in malaria incidence in highlands of Ethiopia and Colombia Science 2014 343 1154 1158 10.1126/science.1244325 24604201

[B61] Caminade C Kovats S Rocklov J Tompkins AM Morse AP Colon-Gonzalez FJ Stenlund H Martens P Lloyd SJ Impact of climate change on global malaria distribution Proc Natl Acad Sci U S A 2014 111 3286 3291 10.1073/pnas.1302089111 24596427 PMC3948226

[B62] Mordecai EA Paaijmans KP Johnson LR Balzer C Ben-Horin T de Moor E McNally A Pawar S Ryan SJ Smith TC Lafferty KD Optimal temperature for malaria transmission is dramatically lower than previously predicted Ecol Lett 2013 16 22 30 10.1111/ele.12015 23050931

[B63] Jetten TH Martens WJ Takken W Model stimulations to estimate malaria risk under climate change J Med Entomol 1996 33 361 371 8667382 10.1093/jmedent/33.3.361

[B64] Dahal S Climatic determinants of malaria and kala-azar in Nepal Reg Health Forum 2008 12 33 37

[B65] Ferguson HM Dornhaus A Beeche A Borgemeister C Gottlieb M Mulla MS Gimnig JE Fish D Killeen GF Ecology: a prerequisite for malaria elimination and eradication PLoS Med 2010 7 e1000303 10.1371/journal.pmed.1000303 20689800 PMC2914634

[B66] Haji KA Khatib BO Smith S Ali AS Devine GJ Coetzee M Majambere S Challenges for malaria elimination in Zanzibar: pyrethroid resistance in malaria vectors and poor performance of long-lasting insecticide nets Parasit Vectors 2013 6 82 10.1186/1756-3305-6-82 23537463 PMC3639098

[B67] Dondorp AM Nosten F Yi P Das D Phyo AP Tarning J Lwin KM Ariey F Hanpithakpong W Lee SJ Ringwald P Silamut K Imwong M Chotivanich K Lim P Herdman T An SS Yeung S Singhasivanon P Day NPJ Lindegardh N Socheat D White NJ Artemisinin resistance in *Plasmodium falciparum* malaria N Engl J Med 2009 361 455 467 10.1056/NEJMoa0808859 19641202 PMC3495232

[B68] Ariey F Witkowski B Amaratunga C Beghain J Langlois AC Khim N Kim S Duru V Bouchier C Ma L Lim P Leang R Duong S Sreng S Suon S Chuor CM Bout DM Ménard S Rogers WO Genton B Fandeur T Miotto O Ringwald P Le Bras J Berry A Barale JC Fairhurst RM Benoit-Vical F Mercereau-Puijalon O Ménard D A molecular marker of artemisinin-resistant *Plasmodium falciparum* malaria Nature 2014 505 50 55 24352242 10.1038/nature12876 PMC5007947

[B69] Diouf G Kpanyen PN Tokpa AF Nie S Changing landscape of malaria in China: progress and feasibility of malaria elimination Asia Pac J Public Health 2014 26 93 100 10.1177/1010539511424594 22087038

[B70] Yin JH Yang MN Zhou SS Wang Y Feng J Xia ZG Changing malaria transmission and implications in China towards National Malaria Elimination Programme between 2010 and 2012 PLoS ONE 2013 8 e74228 10.1371/journal.pone.0074228 24040210 PMC3767829

[B71] Tobgay T Torres CE Na-Bangchang K Malaria prevention and control in Bhutan: successes and challenges Acta Trop 2011 117 225 228 10.1016/j.actatropica.2010.11.008 21114957

