# Supplementary figures and images for: Malaria control in Nepal 1963–2012: challenges on the path towards elimination
Source: Malar J. 2014 Jun 23;13:241. doi: 10.1186/1475-2875-13-241 (PMC4078365; doi:10.1186/1475-2875-13-241)

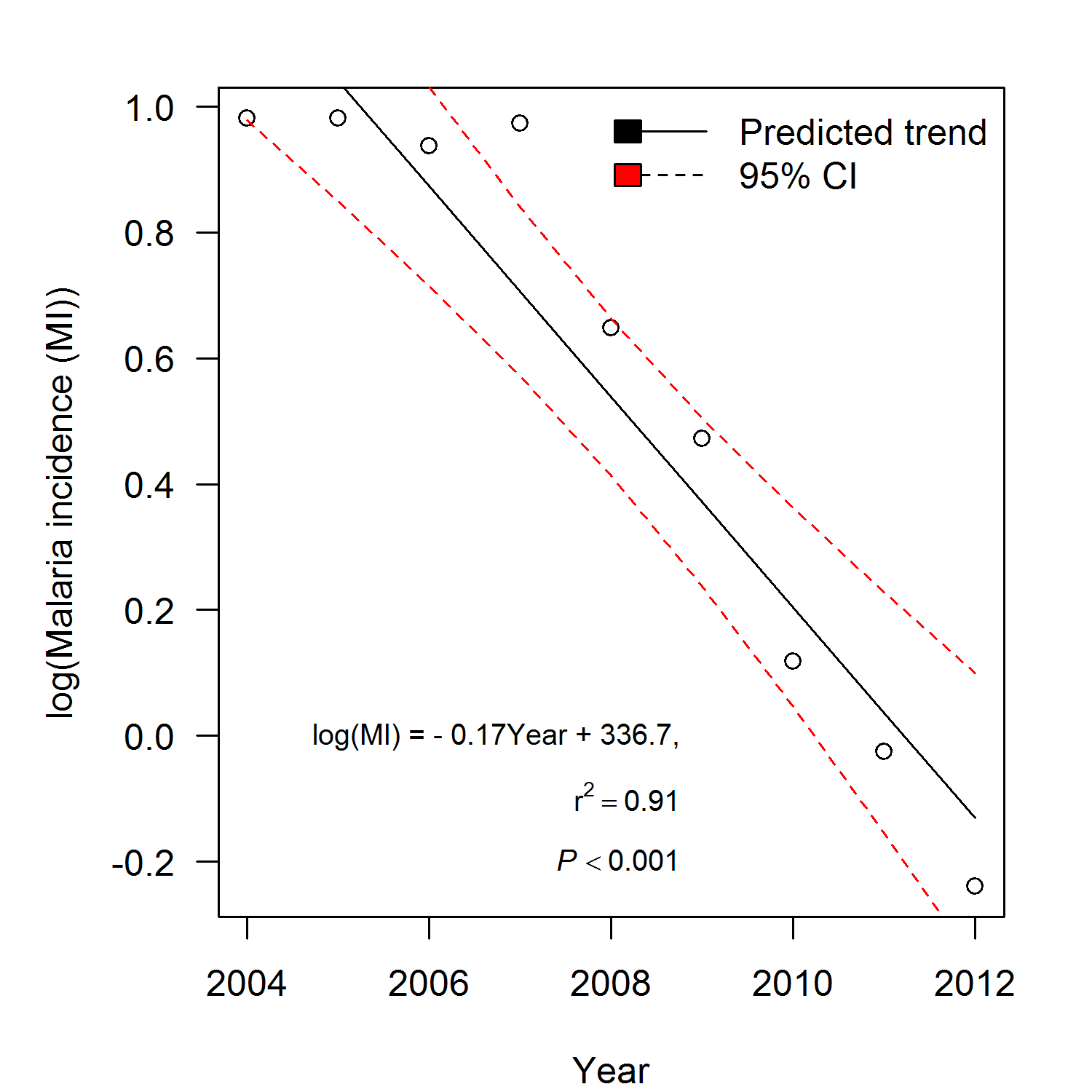

Supplement: Additional file 1 — Linear trend of confirmed malaria incidence in 31 malaria risk districts (2004–2012). The linear regression of confirmed malaria incidence shows significant decline between 2004 and 2012 in 31 malaria-risk districts which accommodate more than 90% reported confirmed cases each year. [file 1475-2875-13-241-S1.tiff]

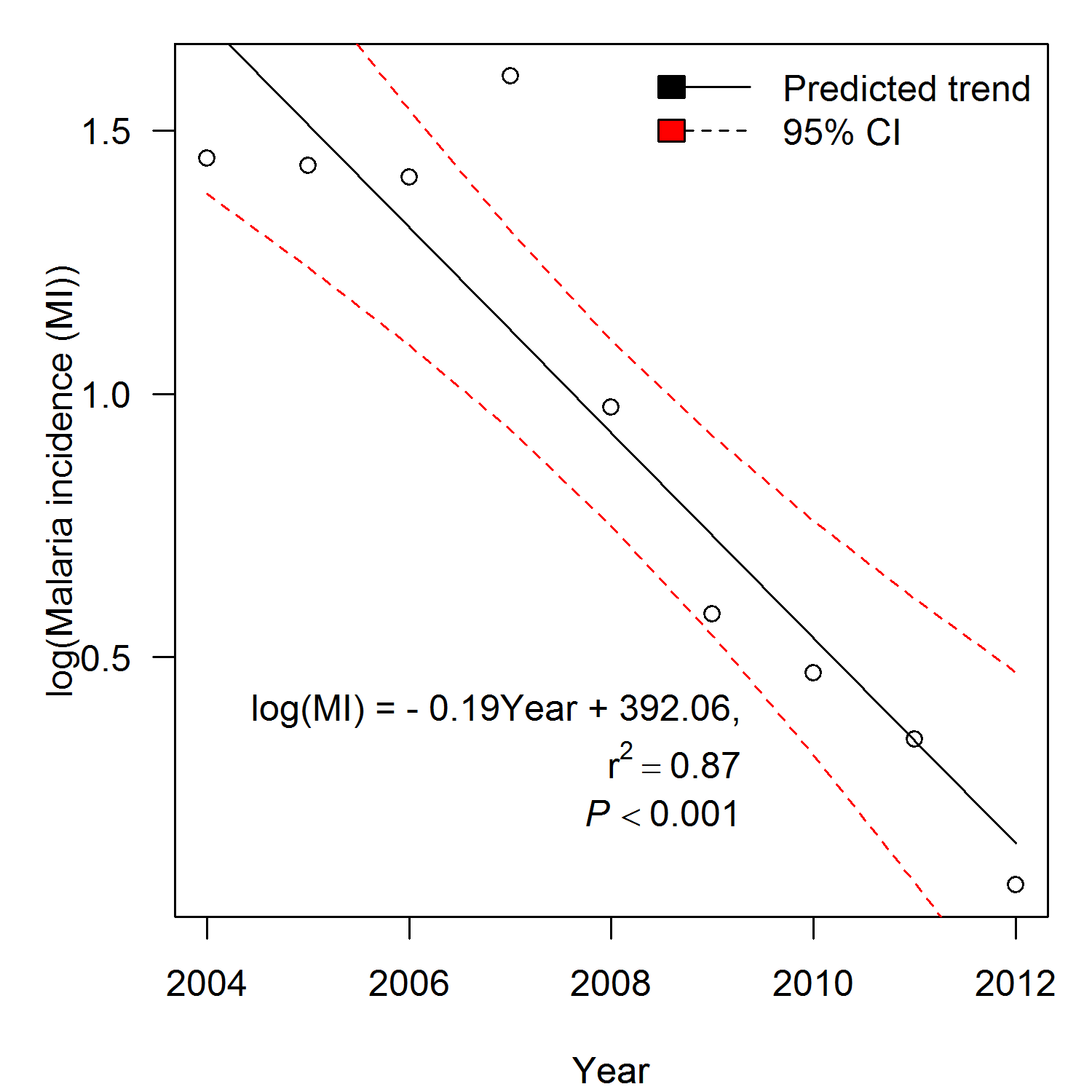

Supplement: Additional file 2 — Linear trend of confirmed malaria incidence in Nepal (2004–2012). The linear regression of confirmed malaria incidence shows significant decline between 2004 and 2012 in Nepal. [file 1475-2875-13-241-S2.tiff]
